# Reflections on kuru

**DOI:** 10.1098/rstb.2008.4024

**Published:** 2008-11-27

**Authors:** Stanley B. Prusiner

**Affiliations:** Institute for Neurodegenerative Diseases, University of California San Francisco513 Parnassus Avenue, HSE-774, San Francisco, CA 94143-0518, USA; Department of Neurology, University of California San FranciscoSan Francisco, CA 94143-0114, USA

My association with kuru came long after the disease had dwindled to fewer than 25 patients annually. By the time I became interested in the disease, cannibalism had ceased (Alpers 1968,1970), and the suggestion had been made that kuru was a transmissible disorder based on its neuropathological similarities with scrapie (Hadlow 1959). The experimental transmission of kuru to non-human primates in 1966 was a seminal finding (Gajdusek *et al*. 1966).

My interest in kuru and scrapie stemmed from caring for a patient dying of Creutzfeldt–Jakob disease (CJD) in the autumn of 1972 during my residency in neurology. Two years later, I set up a laboratory with the aim of purifying the scrapie agent and determining its molecular composition. Once the work was underway, I began to explore the possibility of visiting Papua New Guinea (PNG) and examining patients with kuru. I was fascinated by the lore surrounding this disease and I wanted to perform my own neurological exams on kuru patients. As a physician, I always learned much more about a disease when I had the opportunity to see patients suffering from the disorder.

My efforts to visit PNG began in 1976 when I asked Carleton Gajdusek to help me arrange a visit. While Gajdusek agreed to do so, his receipt of the Nobel Prize in that autumn curtailed these arrangements (Gajdusek 1977). Once I realized that Gajdusek was far too busy to help, I wrote to Michael Alpers and asked him for assistance.

Alpers responded immediately and organized my first visit to PNG in May 1978. Although Alpers was in China when I arrived in Goroka, his former wife, Wendy, was kind enough to meet me. After a couple of days in Goroka, we drove to Purosa on pretty muddy roads. From there, we hiked to Agakamatasa at the southern border of the kuru region where Gajdusek was waiting to welcome me (figure 1**). After nearly a week in Agakamatasa, I set out to examine people with kuru with my guides Anua and Auyana; they are pictured in white shirts in figure 2**. During the next two weeks, I had the opportunity to examine eight patients with kuru.

As Richard Hornabrook had clearly documented, I found that kuru caused cerebellar dysfunction (Hornabrook 1968). Early cases showed a broad-based gait and all of the patients used a walking stick to aid their balance (figure 2**). On neurological examination, they exhibited ataxia of the upper and lower extremities as well as marked intention tremors (figure 2**). Advanced cases manifested profound truncal ataxia. In order to sit or stand, such patients needed to stabilize their bodies by holding onto a stick that was firmly implanted into the ground (figure 3**). While patients with advanced kuru generally answered my questions that were translated by Anua and Auyana, it was clear that they suffered from dementia. All of the patients with advanced kuru exhibited frontal lobe release signs, including snout reflexes (figure 3**), hand grasp (figure 3**) and foot grasp (figure 3**). I had difficulty appreciating the emotional lability often ascribed to people with kuru.

When I left Goroka I stopped in Port Moresby, where I had the opportunity to meet Jettie and Vin Zigas. Vin had been an Australian medical officer in the highlands and was the first physician to study kuru in some detail (figure 1**). In 1957, he was joined by Gajdusek and, together, they wrote two landmark papers on the disease ([Bibr bib4Q7][Bibr bib10Q7]). I chose not to discuss with Vin my clinical findings indicating that kuru patients did not show signs of parkinsonism as he and Gajdusek had thought when they first described kuru. I rapidly developed a warm friendship with Vin and Jettie.

When I returned to New Guinea in 1980, I had the pleasure of meeting Michael Alpers, which marked the beginning of a long friendship. As with my first trip, my guides were Anua and Auyana. Together we saw an additional seven patients whose clinical examinations confirmed my previous impressions. Kuru was a cerebellar disease that eventually progressed to involve other parts of the central nervous system.

Alpers and I thought that it might be nice to write a clinical paper describing my findings. The paper was not ready until the spring of 1982, since my time was consumed by studies in my laboratory on the purification of the scrapie agent.

Preparation of the kuru paper coincided with my introduction of the prion concept in the spring of 1982 (Prusiner 1982). So in the kuru manuscript, I used the term ‘prion’ when I described the unusual properties of infectious pathogens causing kuru, CJD and scrapie. Alpers thought that Gajdusek should be an author, so I added his name to the byline, not anticipating what was to come. Once Gajdusek read the paper, he indicated to me his displeasure with the use of the word prion. When I argued about his objection, Gajdusek contended that using prion would mean that he approved of the word, and he did not. After some discussion, Alpers and I decided that I should back down, and so the word prion appears nowhere in the paper (Prusiner *et al*. 1982).

The kuru story is a legend in the history of medicine. It opened new vistas in the study of neurodegeneration, and it stimulated many people to read about exotic tropical diseases. The disappearance of kuru is a wonderful event in the lives of many people living in the New Guinea highlands.

## Figures and Tables

**Figure 1:**
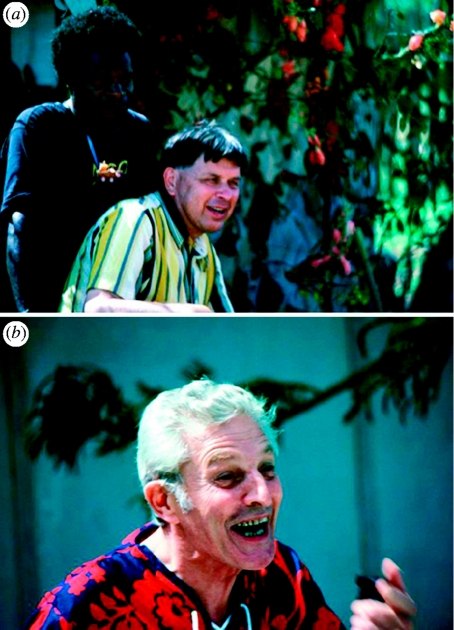
Papua New Guinea, 1978. (**) Carleton Gajdusek in Agakamatasa in 1978, (**) Vin Zigas in Port Moresby.

**Figure 2:**
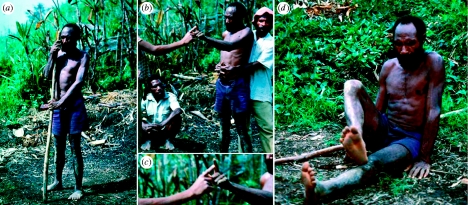
A man with early stage kuru. (**) His gait is broad-based and he is holding his walking stick. (**) The patient shows difficulty with finger–nose testing. He is being held by one of my guides, Anua. To the left, my other guide, Auyana, is crouching. (**) Enlargement of panel (**), patient unable to touch the tip of my index finger. (**) The patient has difficulty with heel–shin testing. Panels (**) and (**), copyright 1982, American Neurological Association; reprinted with permission of John Wiley & Sons, Inc.

**Figure 3:**
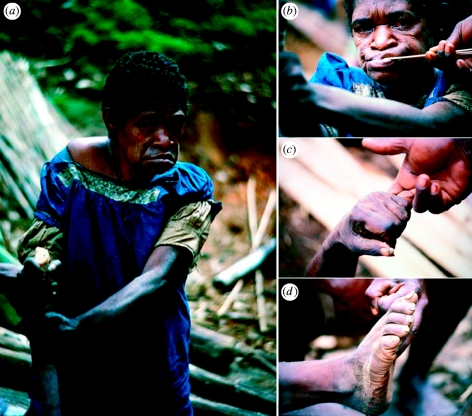
A woman with advanced kuru. (**) For stability, the patient holds a steel rod implanted in the ground. She shows frontal lobe release signs, including (**) snout reflex, (**) hand grasp and (**) foot grasp. Panels (**–**), copyright 1982, American Neurological Association; reprinted with permission of John Wiley & Sons, Inc.
